# The Application of Paclobutrazol to GA_3_-Treated Seed Tuber Potato Fields Does Not Shorten the Growth Cycle or Mitigate Tuber Elongation

**DOI:** 10.3390/plants13162327

**Published:** 2024-08-21

**Authors:** Samuel D. Nomo, Aeden Shlebe, Shimon Rachmilevitch, Akiva Shalit-Kaneh

**Affiliations:** 1 Department of Vegetables and Field Crops, Institute of Plant Sciences Agricultural Research Organization, Gilat Research Center, M.P. Negev, Gilat 85280, Israel; nomosam@post.bgu.ac.il (S.D.N.); aedenshlebe@gmail.com (A.S.); 2 The Albert Katz International School for Desert Research, The Jacob Blaustein Institutes for Desert Research, Sede Boqer Campus, Ben-Gurion University of the Negev, Sede Boqer 849900, Israel; 3 French Associates Institute for Agriculture and Biotechnology of Drylands, The Jacob Blaustein Institutes for Desert Research, Sede Boqer Campus, Ben-Gurion University of the Negev, Sede Boqer 849900, Israel; rshimon@bgu.ac.il

**Keywords:** potato, seed tubers, gibberellic acid, paclobutrazol, growth cycle

## Abstract

Potato seed tubers are a valuable product in potato agriculture. Over the years, studies have been conducted to increase the fraction of mid-size tubers, which are used as a planting material, within the general pool of tuber sizes. Gibberellic acid has been a central component of such studies and has successfully increased the seed-size pool. However, in many cases, misshapen tubers were formed, and the practice has not become widespread. The use of the gibberellic acid inhibitor paclobutrazol has been examined for its ability to increase seed tuber number and tuber growth and to overcome the heat inhibition of tuberization in warm climates. Paclobutrazol has been shown to increase tuber yield and growth rate. In this study, we aimed to test whether the combination of gibberellic acid and paclobutrazol can increase the seed tuber pool, reduce the number of misshapen tubers, and shorten the growth cycle, thus avoiding end-of-season elevated heat conditions and reducing agricultural inputs. Our findings suggest that gibberellic acid on its own can lead to an increase in the number of seed tubers at earlier stages of growth; however, the sequential addition of paclobutrazol was not able to drive even earlier growth or lower the number of misshapen tubers.

## 1. Introduction

Potato (*Solanum tuberosum*) is the fourth largest source of calories [1] and the first-ranked non-cereal crop grown and consumed globally. Additionally, it is a source of income for a very large number of people worldwide who engage in its cultivation and other roles along the value chain. In recent years, potato production has grown greatly in Asia and Africa [2], and China now leads world production, closely followed by India [3]. In Israel, potato is one of the most important crops, and it follows a two-season system. Of the potatoes produced, some 40% are exported, mainly to Europe, while the remaining potatoes are locally used [4]. The first season, fall, typically commences in October or November and ends in March or April, which aligns with relatively cooler weather that is conducive for good potato growth and yield. During the fall crop cycle, plants escape the heat-stress growing conditions which occur toward summer; therefore, yields are expected to be high with good management practices.

The second season, which is spring, starts in December or January, and ends in early or late May [5]. This season can be challenged by unpredictable heat waves and overall higher temperatures occurring in late spring just prior to harvest. This potentially makes potato growth and yield less productive in the second season. The spring crop is used for both local consumption and to obtain seed tubers as a planting material for fall-season planting, while the fall-season produce is for export and local consumption.

Potato tubers are formed at the tips of modified underground stems called stolons [6]. Stolons are formed 2–3 weeks after planting, depending on the variety [7]. The stolon will then grow and elongate due to high gibberellic acid levels and additional signals until it receives signals that induce tuber formation [6]. The tubers will then form just below the growing tip of the stolon and start expanding and accumulating starch, proteins, and additional materials, which serve their role as vegetative reproductive organs [8].

Potato tubers are continuously formed after tuber induction and, therefore, are of varying sizes. Among these different sizes, mid-sized tubers are used as ‘seed tubers’ for planting. Seed tuber sizes range from 35 mm to 60 mm in width (for round tuber varieties), which can be divided into three categories: 35–45 mm, 45–55 mm, and 55–60 mm. The choice of seed tuber size is imperative for successful production and yield as it affects crop establishment, including the rate of stem emergence, the number of stems, development, and growth, influencing its performance in the field [9].

With respect to mechanized planting, seed tuber size may also play a role. Larger and longer tubers may be extracted from the planter more slowly due to higher friction, which could affect the spacing of seed tubers in the field, thus affecting the total plant population per area and yield. Larger tubers may also be more vulnerable to disease due to their large surface area being exposed to disease and physical damage during and after planting them in the soil and placing them in storage. Therefore, the choice of seed tuber size is an essential step for successful production.

Seed tubers are of high value for farmers, and methods to increase the number of seed tubers within the general pool of tuber sizes would be greatly beneficial. Over the years, several strategies have been tested. The use of plant growth regulators, such as the hormone gibberellic acid and its inhibitor paclobutrazol, in potato research have long been studied and their roles are well-defined. Gibberellic acid (GA) is a plant hormone that is responsible for cell elongation, breaking seed and bud dormancy, and increasing branching and stem elongation [10]. GA’s role in tuberization, the initiation of tubers, is repressive in nature, and the exogenous application of GA delays tuber formation and growth [11] and enhances canopy growth [12]. GA application has been shown to increase the number of medium-sized tubers that are 28–45 mm in diameter through shifting the tuber size distribution to smaller sizes due to tuberization delay and slower tuber growth [13]. GA also influences stolons by elongating their growth and inducing more stolon branching potential, providing more starting points to initiate tuberization [13,14]. Additionally, GA has been shown to reduce dry matter content [15], and early GA application has been shown to cause the formation of elongated tubers, which are viewed negatively with respect to visual esthetics [10,13]. Trials using GA_3_ to manipulate stolon formation, tuber growth, and tuber size distribution have been conducted with applications to pre-planted tubers, the soil of growing plants, and the foliage of growing plants [16].

Paclobutrazol (PBZ), an inhibitor of gibberellin, is a triazole derivative that also elevates levels of other phytohormones, cytokinin, and abscisic acid (ABA), and increases the photosynthetic rate to impact plant growth and development [17]. Paclobutrazol impedes ent-kaurene oxidase activity. This is an enzyme in the GA biosynthetic pathway that catalyzes the oxidation of ent-kaurene to ent-kaurenoic acid, a precursor of GA [17,18]. PBZ serves as a structural enantiomer, with R and S configurations at the two chiral carbons bearing the hydroxyl groups [18] known to inhibit GA biosynthesis, and it is used as a growth retardant and stress protectant [19,20,21]. The external foliar application of PBZ is known to alter plant vegetative growth, causing shorter plant height, thick dark leaves, and a reduced leaf area. In wheat, it is noted for grain yield improvement and thermo-tolerance through another mechanism, and not the GA inhibition mechanism [22]; however, for potato tubers, it is reported to increase tuber size and weight, as well as dry matter content [23,24,25]. PBZ achieves this through partitioning more photosynthetic assimilates to the tubers via hormonally regulated changes in the sink-to-source process at the expense of the canopy. This ability of PBZ to manipulate tuber growth is achieved by interfering with GA levels. This has led many researchers to suggest PBZ as a plant growth regulator that is able to regulate tuber development and growth in warm climates, in which GA levels are elevated due to heat [25,26]. The application of PBZ has been consistently shown to reduce the total tuber number while increasing the total tuber weight; however, there are also reports that foliar application of PBZ can increase the seed tuber number when applied to more densely planted fields [23].

Previous studies have used GA_3_ and PBZ combinations in different crops, including potato; however, these applications were not both foliar. For example, dipping pre-planted tubers in GA_3_, in order to increase stem emergence and stem number, was complemented with early PBZ foliar application in the potato variety Spunta [23] and, in carrot, PBZ was applied using the soil drench method [27]. In Israel, GA_3_ and PBZ are not used in potato agriculture. In the past, tests have been carried out in Israel with GA_3_, but they have not been further pursued due to the formation of misshapen tubers (personal communications).

In this study, we used GA_3_ and PBZ as single and sequentially combined plant growth regulators in agricultural field settings to investigate the effects of the exogenous application of GA_3_ and PBZ to regulate tuber development, growth, and size. PBZ was added at four different times to GA-treated fields to speed up tuber growth for a possible shorter plant growth cycle and a reduction in misshapen tubers. A shorter cycle will also lead to a decrease in agricultural inputs, such as water, fertilizer, and pesticides, and help in avoiding the elevated temperatures toward the end of the spring season. Our findings revalidate GA’s capability in increasing the seed tuber number and show it can also shorten the growth cycle. However, the sequential application of GA_3_ and PBZ did not shorten the growth cycle further, nor did it mitigate the misshapen tubers formed by GA_3_ application.

## 2. Results

### 2.1. Effects of Sequential GA and PBZ Application on Potato Canopy

**Figure 1:**
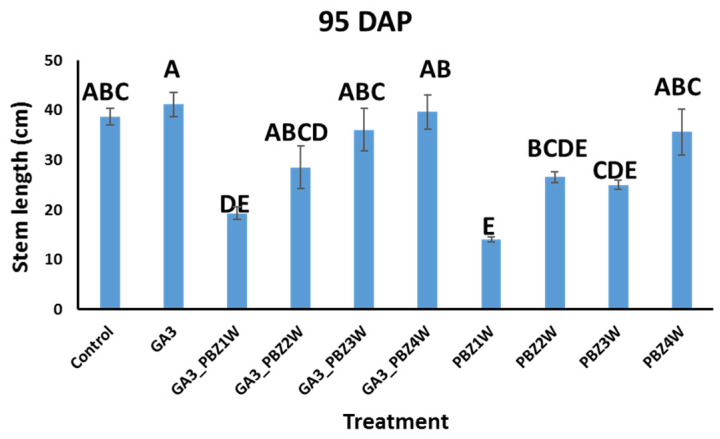
Effects of GA_3_ and PBZ application on Sifra cultivar stem length at 95 DAP. Different letters indicate statistically significant differences (ANOVA, Tukey–Kramer’s HSD, *p* value ≤ 0.05). Data are from Experiment 1. Weeks 1 to 4 are denoted as 1W to 4W.

To test our ability to simultaneously increase the seed tuber number and shorten the growth cycle, we carried out a field experiment in the spring of 2022: Experiment 1 with the Sifra variety, onto which we sprayed GA_3_ [13] (see Section 5 and Figure S2). We then sprayed PBZ onto the GA_3_-treated plants at four different times, weeks 1–4 (Figure S1). We reasoned that if PBZ would have a transient effect on increased tuber growth, in this way, we would find the optimal time to apply it. As a control, we sprayed untreated Sifra plants with PBZ at the same times. To characterize the effects of our treatments on the plants, we measured several canopy growth parameters, including the stem length (Figure 1), canopy dry weight, and stem number at 95 DAP (Figure S3). Of these parameters, the only one that showed statistically significant differences was the stem length. PBZ application showed a clear, significant, and expected dwarfing effect on the plants treated with it; however, the later the application in the growth cycle, the smaller the effect on stem length shortening. Sequential combinations of GA_3_ and PBZ again showed dwarfing effects. However, comparing the corresponding treatments of PBZ with and without a previous GA_3_ treatment showed that the GA_3_ pre-treatment reduced the ability of PBZ to take effect, although this was only statistically significant in the sequential GA_3_–PBZ1W vs. PBZ1W and the GA_3_–PBZ3W vs. PBZ3W single treatments (Figure 1), when comparing them as pairs (Student’s *t*-test, *p* value ≤ 0.05). The dry canopy weight and stem number did not differ between the treatments (Figure S3).

### 2.2. Effects of Sequential GA and PBZ Application on Potato Tubers

To test the effects of our treatments on the seed tuber number, we sampled tubers in the field at 95 DAP and 102 DAP. At 95 DAP, differences in the seed tuber numbers were not significant via stringent ANOVA analysis; however, compared to GA_3_, GA_3_–PBZ3W, GA_3_–PBZ4W, and PBZ2W all had significantly higher numbers of tubers (Student’s *t*-test, *p* value ≤ 0.05), whereas GA_3_–PBZ1W and GA_3_–PBZ2W did not (Table 1). We took this result as an indication of possible merit regarding the combined use of sequential GA_3_–PBZ applications at the later times of weeks 3 and 4. One week later, at 102 DAP, there were no significant differences. In terms of the percentage of the total number of tubers at 102 DAP; GA_3_ and GA_3_–PBZ3W had a significantly higher percentage compared to PBZ2W.

To gain additional insight into the effects of our GA_3_ and PBZ treatments on the tubers, we dehaulmed the plants (i.e., terminated tuber growth by removing the canopy) at three different times. We counted the seed-size tuber numbers, calculated the seed-size tuber percentage, and measured the tuber length of the seed-size category at 106, 113, and 121 DAP (Table 2). There were no significant differences in the seed-size tuber numbers at any of the dehaulming times. Later dehaulming times had lower tuber numbers overall, indicating growth times that were too late for the collection of seed tubers. Looking at the percentages at 106 DAP, the GA_3_ treatments had a significantly higher percentage of seed-size tubers at 80% vs. PBZ4W at 65%. We next looked at tuber length. One issue reported for GA_3_ treatments in the past was elongated and misshapen tubers. We wanted to test whether sequential GA_3_ and PBZ treatments would reduce this effect. GA_3_ did not form significantly longer tubers vs. the control or PBZ treatments, regardless of the dehaulming times in Experiment 1 (Table 2). However, in the sequential GA_3_–PBZ treatments, the earlier PBZ applications at GA_3_–PBZ1W or GA_3_–PBZ2W were significantly longer at 113 days vs. PBZ1W and PBZ2W, indicating that PBZ did not mitigate tuber elongation (Table 2).

### 2.3. Progeny of GA PBZ Sequential Application

In order to test the efficiency and quality of seed tubers produced by our different treatments, we conducted a progeny test in which we used seed tubers that we produced and planted in a field experiment in the following fall season (2022). We measured the weight, number, and length of the tubers in the marketable sizes of 35–80 mm to determine differences. No significant differences were detected in the tuber weight or tuber number (Table 3). Additionally, no significant differences were identified in the lengths of these tubers, which were the second generation after our treatments (Table 4).

### 2.4. Examination of Earlier Dehaulming and Increased PBZ Concentrations on Seed Tuber Number, Growth Cycle Length, and Dry Matter Content

After identifying a potential increase in the seed tuber size number in the later sequential GA3–PBZ combinations of 3W and 4W at 95 DAP in Experiment 1 (Table 1), we decided to repeat the experiment on a larger scale and dehaulm the plants earlier to test for a possible shorter cycle effect; we termed this Experiment 2. We conducted a similar experiment to Experiment 1 (Figure S1) in the spring of 2023, this time with sequential applications of GA_3_–PBZ 1W and GA_3_–PBZ4W and with PBZ at the same and a higher concentration to test for improved performance. One central difference was the planting density which, in 2023, was intended for market tubers and not seed tubers. However, we reasoned that we would still be able to test the benefits of our treatments (see Section 5, Experiment 2, and Section 3). The dehaulming times were 82, 95, and 103 DAP. In all cases, the GA_3_ treatments produced the highest number of tubers and the highest weight in the seed-size categories, outperforming the control with statistical significance with few exceptions, and followed closely by the sequential treatments of GA_3_–PBZ (Table 5, Tables S1 and S2). In all cases, the sequential GA_3_ PBZ applications did not significantly increase the tuber number or weight. When looking at the tuber length of the seed-size tubers in our different treatments (Table 6), we saw more clearly and significantly than in our previous experiment (Table 2) that the GA_3_ treatments formed significantly longer tubers than the control and single PBZ treatments. However, the sequential GA_3_–PBZ treatments did not significantly shorten the tubers vs. GA_3_ alone.

## 3. Discussion

In our study, we combined foliar applications of GA_3_ and PBZ. GA_3_ served to increase the seed tuber number through lowering the growth rate of the existing tubers and delaying the initiation of new tubers, which resulted in fewer large tubers [13]. In certain cases, additional initiation points for tuberization are formed by stolon branching, due to a loss of apical dominance in the stolon tip [13]. We then supplemented the GA_3_-treated plants with PBZ, which can speed up the rate of tuber filling [25,28], in order to test whether we could attain tubers of the desired seed tuber size earlier in the growth cycle. A positive result would benefit growers through reducing agricultural inputs. Additionally, we tested to see whether the misshapen tubers reported in the use of GA_3_ with respect to length could be mitigated through the addition of PBZ. We also tested whether GA_3_ and PBZ treatments effected the efficiency of the seed tubers when planted in the next season. Of the canopy parameters that we measured, the clearest effects of GA_3_ and PBZ were on the stem lengths. PBZ-treated plants had significantly shorter stems (Figure 1), and this effect was stronger the earlier the PBZ was applied, as shown previously [24]. In the sequential GA_3_–PBZ applications, the effect of the earlier PBZ application was consistent in its ability to shorten the stem lengths; however, the initial application of GA_3_ seemed to counter the effectiveness of the PBZ. This was statistically significant, according to the Student’s *t*-test, in the cases of GA_3_–PBZ1W vs. PBZ1W and GA_3_–PBZ3W vs. PBZ3W (Figure 1). A possible explanation for this intermediate result when combining both GA_3_ and PBZ is the nature of PBZ activity [17,18]. PBZ inhibits GA biosynthesis and not its downstream signaling; however, GA metabolism is highly complex. GA_3_ applied to the canopy can work locally in the leaves and stems; however, its systemic below-ground effect is not direct. Locally, GA_3_ increases canopy growth at the expense of tubers, altering sink–source photoassimilate partitioning. GA_3_ alters GA metabolism in the canopy and affects other hormones as well [27]. Systemic GA effects are thought to be mainly relayed by GA_12_ and GA_20_, which reach their destination and are metabolized to active GA forms, namely, GA_1_, GA_3_, GA_4_, and GA_7_ [28,29]. The biosynthesis of these systemic forms of GA can be affected through PBZ application, thus modulating GA systemic movement. Additionally, PBZ itself has been shown to move systemically in several plants and can, therefore, have systemic effects [30]. The gradual reduction in the effect of GA_3_–PBZ on stem length in the later application times of 3W and 4W was also due to the growth stage the plant had reached at the time of application. Considering the properties of tuber shape, we compared the lengths of the treatment-produced tubers, which would serve as seed tubers (Table 6). Tubers treated with GA_3_ alone were longer than those from the control. Tubers treated sequentially with GA_3_ and PBZ did not significantly mitigate the GA_3_ effects. Studies on tuber formation in vitro and in vivo have suggested that the swelling of the tuber begins at the older internodes formed at the stolon tip [31]. For example, a 5 mm stolon tip could contain eight internodes, each of which would form a meristem or eye later in tuber formation. The first formed node, node 1, is at the part farthest away from the tip, while the last one formed, node 8, at the very tip, will form the apical meristem of the tuber. The stolon will begin swelling at the upper part of the first internode or another of the first formed internodes. When this process starts, the cells expand in size and then divide longitudinally until the tuber is around 0.8 cm in diameter, at which time randomly oriented divisions occur, driving growth. It may be the case that the application of GA_3_, which is known to cause stolon elongation, could already affect the tubers, which would be formed in the future from these stolons by changing the distances between these nodes at the stolon tip. Beyond this, GA_3_ could also change the cell divisions of tubers already initiated. This suggests that it would be difficult to rescue GA_3_-driven tuber elongation as it happens at very early stages, potentially even before tuberization. We next tested whether the seed tubers formed in Experiment 1 had any reduced ability to perform as seed tubers. We planted them in the fall season of 2022 after cold storage and measured the tuber weights and numbers in plots planted with the seed tubers from our different treatments in the spring of 2022. Testing the marketable sizes, 35–80 mm in width, we found no reduction in tuber weight or number or increase in length (Table 3 and Table 4). To confirm the initial indications in Experiment 1 that the sequential application of GA_3_ and PBZ at four weeks was beneficial to increasing seed tuber number (Table 1, 95 DAP), we repeated our field experiment at a larger scale in the spring of 2023, Experiment 2. In this experiment, we also included treatments with higher concentrations of PBZ. Our earliest dehaulming was 82 DAP to test for a shortened growth cycle. Our results showed that GA_3_ had the greatest number of seed tubers already at 82 DAP, followed closely by GA_3_–PBZ4W (Table 5). However, only the results for GA_3_ vs. control had statistical significance. A central difference between our 2022 and 2023 experiments was in the spacing or density of planting. In 2022, the density was seven tubers per 1 m, which is standard for seed tuber production as the competition between the plants causes the formation of more mid-sized tubers [32]. In 2023, the planting density was 3.7 seed tubers per 1 m, the density used for market tubers. We reasoned that, although the comparison was not optimal, the overall positive or negative effects of our treatments should be comparable by looking at the differences between the treatments within each year. Additionally, the treatments in 2023 suggest that a field planted initially for fresh market tubers could be converted into a seed tuber-designated field. Finally, it is important to note that PBZ is a triazole, similar in structure to chemicals that have effects as endocrine disruptors, thus requiring careful consideration of its use [33].

## 4. Conclusions

Our sequential foliar GA_3_–PBZ treatments were not found to be capable of shortening the growth cycle when aiming for specific seed tuber sizes, while the GA_3_ treatment alone was effective in this regard. Foliar PBZ was also not able to mitigate the extended tuber-length effects of GA_3_. GA_3_–PBZ combinations did not reduce the efficiency of the formed seed tubers when they were planted in the field in a progeny test the following season. Based on our specific experimental setup and variety tested, we would not recommend the GA_3_–PBZ sequential treatments that we studied as an agricultural practice. However, future studies considering additional parameters may have merit. Potato varieties can be categorized roughly into early, mid, and late maturation, which are measures of the time from tuber initiation to tuber maturity and the beginning of leaf senescence. It would be beneficial in future studies to assess the optimal times of GA_3_ and PBZ application with respect to these genetically based traits in different potato varieties.

## 5. Materials and Methods

### 5.1. Plant Material and Growth Conditions

Two close fields were chosen near Kibbutz Sa’ad, Israel, for the spring 2022 (31°27′43.1″ N 34°33′20.3″ E) and the spring 2023 (31°27′00.2″ N 34°33′46.1″ E) hormonal treatments within commercial plots. These experiments were termed Experiments 1 and 2, respectively. This area is defined with a semi-arid climate, with a mean annual temperature of 20.1 °C, mean annual air humidity of 68%, and annual precipitation of 380 mm (according to agrometeorological reports from the Ministry of Agriculture, Israel). The irrigation and fertilization regimes and the agro-techniques in the field were those of the commercial grower. Triple super phosphate (46%) at 240 kg/ha and KCl in powder form at 170 kg/ha were applied before planting while preparing the hills. A total of 350 kg/ha of nitrogen was applied via irrigation from full plant emergence until approximately one month before dehaulming. Water was given through sprinkler irrigation at a total of 3580 mm/ha. Watering was administered, on average, every three days with an amount of 150 mm/ha, along with 2–3 kg/ha of nitrogen. After dehaulming, watering was given at 50 mm/ha every other day to cool the soil until harvest.

### 5.2. Experiment 1, Spring 2022

Ten hormonal treatments (graphically presented in Figure S1) were assessed in this experiment. Each treatment was replicated three times. Each plot consisted of three ridges (6 m) over 5 m for 30 m^2^. Therefore, three replicates per 90 m^2^ area with 10 treatments resulted in a total of 900 m^2^. The treatments consisted of a control, GA_3_ as a one-time application to the plants (53 days after planting), and four combinatorial treatments of GA_3_ and PBZ, where PBZ was applied to plants at week 1 (60 DAP), week 2 (67 DAP), week 3 (74 DAP), and week 4 (81 DAP) after GA_3_ application. Therefore, the treatment names were denoted as GA3–PBZ1W, GA3–PBZ2W, GA3–PBZ3W, and GA3–PBZ4W. The other four treatments included PBZ-only controls, applied at the same time with the four PBZ combined treatment times, and were named PBZ1W, PBZ2W, PBZ3W, and PBZ4W. The GA_3_ treatment was 0.327 g/L Giberllon^TM^, obtained from Gadot Agro, Givat Brenner, Israel. The treatment also contained the surfactant Triton^®^ X-100 (Sigma-Aldrich, Merck KGaA, Darmstadt, Germany), at 0.025% (*v*/*v*). The PBZ treatment was 0.09 gr/L of Cultar^TM^ (ADAMA, Ashdod, Israel). The treatment also contained the surfactant Shatah90^TM^ (ADAMA, Ashdod, Israel) at 0.1% (*v*/*v*). Treatments were applied as foliar spray via a motorized backpack sprayer in the spring of 2022. The GA_3_ treatment was applied when plants had, on average, one 0.5 cm diameter tuber (Figure S2) and good plant emergence (approximately 90% emergence of seed tubers from the soil). The Sifra variety was chosen, as it is very commonly used. Sifra seed tubers of 35–45 mm were mechanically planted in the spring of 2022 at 3480 kg/ha in Loess soil, with 7 tubers per 1 m at a spacing of 14.2 cm for seed production. The planting date was 29.12.21. The plants were sampled manually at 95 DAP and 102 DAP by extracting three plants in three replicates, for a total of 9 plants per treatment. Each plant was defined as consisting of all the tubers made from the stems originating from one mother tuber. The plants were dehaulmed on three dates, 15.4.22 (106 DAP), 22.4.22 (113 DAP), and 30.4.22 (121 DAP), and later manually extracted. This was carried out manually by cutting the canopy of 1 m^2^. Harvesting was performed manually on 27.5.22. Size sorting and weighing was achieved via an optical sorting system.

### 5.3. Experiment 2, Spring 2023

Experiment 2 was also carried out near Kibbutz Sa’ad (31°27′00.2″ N 34°33′46.1″ E). It was a follow-up experiment to investigate the findings from Experiment 1. The field was planted on 25.1.23. The tubers planted were of the Sifra variety, sizes 45–55 mm. A total of 3018 kg/ha were planted, with an average tuber weight of 84 g and at spacings of 3.7 seed tubers per 1 m (27 cm) for market-sized tubers. The experiment consisted of ten treatments: control, GA_3_ 0.327 g/L, GA_3_ followed by PBZ 0.09 g/L one week after, GA_3_ followed by PBZ 0.27 g/L one week after, and GA_3_ followed by PBZ four weeks after, with either PBZ 0.09 g/L or PBZ 0.27 g/L. These treatments were complemented with PBZ-only control treatments, which were applied at the same time as the combined PBZ applications at one week and four weeks after GA_3_ application. The treatments were named control, GA3, GAPBZ90–1W, GAPBZ270–1W, GAPBZ90–4W, GAPBZ270–4W, PBZ90–1W, PBZ270–1W, PBZ90–4W, and PBZ270–4W. The commercial products used for the application, plot sizes, and replication were the same as in Experiment 1. The application time was also the same as in Experiment 1. Mechanical harvesting was conducted on one ridge (2 m by 4 m, for 8 m^2^) per replicate. Dehaulming was performed using mechanized canopy removal at 82, 95, and 103 DAP. The mechanical harvesting of these plots was performed on 28.5.23 (123 DAP). Size sorting and weighing was carried out using an optical sorting system.

### 5.4. Progeny Test

This experiment tested only tubers from Experiment 1 and was conducted near Moshav Nave in the Negev, which has sandy soil (31°10′18.2″ N 34°21′07.3″ E). Seed tubers harvested from Experiment 1 were maintained in the Atzmona commercial cold storage facility close to Moshav Nave from late May to November of 2022. The tubers were then subjected to an efficiency and quality analysis to determine whether the previously applied treatments would affect the next generation in terms of plant performance, tuber growth, development, and quality. The tubers were manually planted on 3.11.22 with a pointed chisel wooden tool to a depth of 15 cm at a spacing of 25 cm. Three replicate plots of 2 m^2^ for each treatment were planted, and the tubers were allowed to grow under the farmers’ growing practices until harvest. The tubers were manually harvested, then sorted using an optical sorter.

### 5.5. Above-Ground Parameters

In Experiment 1, the stem length, stem number, and canopy dry weight were measured and documented at 95 DAP. Three plants per replicate (three replicates, for a total of nine plants) were manually extracted per progeny of a specific treatment from the soil with a garden fork. A plant was defined as all the plant material emerging from a single mother tuber.

### 5.6. Below-Ground Parameters

In Experiment 1, the tubers were collected from the plants, as described above, at 95 DAP and 102 DAP. The tubers that were formed and newly forming (from 0.5 cm in diameter) were meticulously collected from each mother tuber, counted, and weighed. Each replicate collection was made up of 3 to 4 mother seed tubers; these were all added and normalized by the number of mother seed tubers to obtain replicate data. The tubers collected in Experiment 1 at 106 DAP and later from 1 m^2^ plots were sorted and weighed with an optical sorter system. The tubers collected in Experiment 2 and the progeny test were also sorted with the optical system.

### 5.7. Data Analysis

Statistical analysis was performed using the JMP statistical package (SAS Institute Inc., Cary, NC, USA). Tukey’s post-hoc HSD test and the Student’s *t*-test were performed to determine the possibility of statistically significant differences between the means of the treatments and the seed tuber categories, tuber yield (in number), and plant canopy parameters. The significant levels were set at *p* < 0.05. The graphs and plots were made using Excel 2016.

## Figures and Tables

**Table 1:** The number of seed-size tubers and percentage of seed-size tubers in the GA_3_ and PBZ treatments. Seed tubers are defined as 35–60 mm wide tubers. The tubers were sampled at 95 and 102 DAP. Different letters indicate statistically significant differences (ANOVA, Tukey–Kramer’s HSD, *p* value ≤ 0.05). The number of tubers is normalized per plant, i.e., the mother tuber. The percentage of seed-size tubers is out of the total number of tubers of all sizes. The results are the means of three replications. The standard error (SE) of the mean is indicated. The data are from Experiment 1. Weeks 1–4 are denoted as 1W to 4W.

		Number of 35–60 mm Tubers						% of 35–60 mm Tubers Out of Total				
Treatments	95 DAP	SE (±)	*p* Value ≤ 0.05	102 DAP	SE (±)	*p* Value ≤ 0.05	95 DAP	SE (±)	*p* Value ≤ 0.05	102 DAP	SE (±)	*p* Value ≤ 0.05
Control	6.02	1.26	a	6.06	0.36	a	61	9.74	a	62	3.28	ab
GA3	4.17	0.51	a *	7.81	0.44	a	57	1.87	a	78	4.05	a
GA3–PBZ1W	5.42	0.63	a	5.9	1.06	a	58	5.34	a	58	2.24	ab
GA3–PBZ2W	5.2	1.29	a	5.45	0.29	a	57	5.51	a	64	5.47	ab
GA3–PBZ3W	6.81	0.51	a *	6.34	0.88	a	62	2.36	a	79	5.05	a
GA3–PBZ4W	7.01	1.02	a *	6.45	1.13	a	60	5.76	a	69	4.40	ab
PBZ1W	4.47	0.70	a	5	1.02	a	55	5.17	a	68	6.14	ab
PBZ2W	6.78	0.91	a *	5.73	0.84	a	68	3.98	a	53	5.02	b
PBZ3W	5.06	0.47	a	5.67	0.19	a	70	7.06	a	69	2.93	ab
PBZ4W	6.64	1.01	a	6.78	0.91	a	72	1.55	a	68	1.88	ab

* Letters with asterisks indicate significant differences between GA_3_ and the other indicated treatments (Student’s *t*-test, *p* value ≤ 0.05).

**Table 2:** Seed-size tuber numbers (35–60 mm in width), percentages, and length in mm. Plants were dehaulmed at three different times: 106, 113, and 121 DAP. The tuber numbers are normalized per mother tuber. Different letters indicate statistically significant differences (ANOVA, Tukey–Kramer’s HSD, *p* value ≤ 0.05). The means are the results of three replications. The standard error (SE) of the mean is indicated. The data are from Experiment 1. Weeks 1–4 are denoted as 1W to 4W.

	Number of Tubers (35–60 mm)								
Treatment	106 DAP			113 DAP			121 DAP		
	Mean	SE (±)	*p* Value ≤ 0.05	Mean	SE (±)	*p* Value ≤ 0.05	Mean	SE (±)	*p* Value ≤ 0.05
Control	6.0	1.0	a	4.9	0.5	a	6.7	0.6	a
GA3–PBZ1W	6.4	0.2	a	5.0	0.2	a	5.9	0.2	a
GA3–PBZ2W	7.4	0.2	a	6.1	0.7	a	6.5	0.9	a
GA3–PBZ3W	6.8	1.0	a	5.0	0.6	a	5.9	0.5	a
GA3–PBZ4W	7.5	0.2	a	5.5	0.4	a	7.3	0.9	a
GA3	7.8	0.1	a	4.7	1.0	a	5.4	0.6	a
PBZ1W	5.9	0.1	a	5.6	0.6	a	5.3	1.0	a
PBZ2W	6.9	0.4	a	6.2	0.7	a	5.5	0.3	a
PBZ3W	5.7	0.4	a	7.0	1.2	a	4.3	0.1	a
PBZ4W	5.7	0.7	a	6.4	na	a	5.4	0.8	a
	**% of 35–60 mm Tubers out of Total**								
**Treatment**	**106 DAP**			**113 DAP**			**121 DAP**		
	**Mean**	**SE (±)**	***p* value ≤ 0.05**	**Mean**	**SE (±)**	***p* value ≤ 0.05**	**Mean**	**SE (±)**	***p* value ≤ 0.05**
Control	69	3	ab	58	4	a	72	4	a
GA3–PBZ1W	73	4	ab	65	5	a	68	3	a
GA3–PBZ2W	75	2	ab	70	2	a	70	6	a
GA3–PBZ3W	71	4	ab	64	1	a	66	3	a
GA3–PBZ4W	77	1	ab	64	1	a	73	2	a
GA3	80	2	a	61	9	a	65	1	a
PBZ1W	74	2	ab	70	4	a	67	7	a
PBZ2W	69	3	ab	63	4	a	65	1	a
PBZ3W	70	2	ab	70	3	a	68	1	a
PBZ4W	65	2	b	67	na	a	67	5	a
	**Tuber Length of 35–60 mm Seed-Size Tubers**								
**Treatment**	**106 DAP**			**113 DAP**			**121 DAP**		
	**Mean**	**SE (±)**	***p* value ≤ 0.05**	**Mean**	**SE (±)**	***p* value ≤ 0.05**	**Mean**	**SE (±)**	***p* value ≤ 0.05**
Control	71.3	0.9	bcde	70.0	0.4	bc	72.7	0.9	bcd
GA3–PBZ1W	75.2	0.6	abcd	74.4	1.5	ab	75.1	1.8	abc
GA3–PBZ2W	77.7	1.3	ab	74.5	1.7	ab	76.1	1.7	abc
GA3–PBZ3W	75.5	1.7	abcd	79.3	2.8	a	79.1	1.0	ab
GA3–PBZ4W	78.0	1.8	a	78.2	2.2	a	80.4	0.9	a
GA3	76.3	1.7	abc	73.2	1.1	ab	77.4	2.3	abc
PBZ1W	69.9	1.2	cde	65.2	1.0	c	67.3	1.4	d
PBZ2W	67.4	0.8	e	65.1	0.1	c	70.3	0.8	cd
PBZ3W	69.5	1.7	de	69.3	1.3	bc	70.9	2.4	cd
PBZ4W	70.9	0.8	cde	na	na	na	71.6	1.2	cd

**Table 3:** Assessing the seed-size tuber efficiency and quality in forming progeny. Seed tubers formed from all treatments in our spring 2022 experiment were used the following fall season as planting material. Progeny tubers were tested for differences in weight (kg) and numbers per 1 m^2^ in the marketable sizes of 35–80 mm. Different letters indicate statistically significant differences (ANOVA, Tukey–Kramer’s HSD, *p* value ≤ 0.05). The means are the results of three replications. The standard error (SE) of the mean is indicated. The data are from the progeny test. Weeks 1–4 are denoted as 1W to 4W.

Marketable Tubers (35–80 mm)						
Treatment	Tuber Weight			Tuber Number		
	Mean (kg)	SE (±)	*p* Value ≤ 0.05	Mean	SE (±)	*p* Value ≤ 0.05
Control	10.7	0.2	a	74.3	6.4	a
GA3	10.4	0.6	a	70.7	7.9	a
GA3–PBZ1W	11.9	0.4	a	92.0	8.5	a
GA3–PBZ2W	11.7	0.5	a	80.3	5.2	a
GA3–PBZ3W	12.1	0.6	a	90.0	4.6	a
GA3–PBZ4W	11.2	0.4	a	82.0	3.5	a
PBZ1W	10.2	0.6	a	72.3	5.7	a
PBZ2W	11.8	0.3	a	89.0	6.0	a
PBZ3W	10.5	1.1	a	71.0	8.7	a
PBZ4W	11.9	1.4	a	86.0	9.3	a

**Table 4:** Testing the tuber lengths of the progeny produced from treatment formed seed tubers. The average tuber lengths (mm) of the width categories of 15–35 mm, 35–60 mm, and 60–80 mm are presented as the results from three replicate plots of 1 m^2^. Different letters indicate statistically significant differences (ANOVA, Tukey–Kramer’s HSD, *p* value ≤ 0.05). The means are the results of three replications. The standard error (SE) of the mean is indicated. The data are from the progeny test. Weeks 1–4 are denoted as 1W to 4W.

Tuber Length of Marketable Tubers									
Treatment	15–35 mm Width			35–60 mm Width			60–80 mm Width		
	Mean Length (mm)	SE (±)	*p* Value ≤ 0.05	Mean Length (mm)	SE (±)	*p* Value ≤ 0.05	Mean Length (mm)	SE (±)	*p* Value ≤ 0.05
Control	31.0	0.9	a	64.1	0.9	a	86.0	1.4	a
GA3	33.6	2.0	a	61.4	1.1	a	86.9	0.9	a
GA3–PBZ1W	32.8	1.8	a	61.6	1.3	a	85.2	1.8	a
GA3–PBZ2W	31.2	0.9	a	64.7	1.0	a	86.1	0.5	a
GA3–PBZ3W	33.2	1.2	a	62.3	0.2	a	84.4	0.6	a
GA3–PBZ4W	32.2	1.3	a	60.8	0.8	a	85.8	1.3	a
PBZ1W	33.1	0.7	a	62.8	0.9	a	85.5	0.5	a
PBZ2W	32.0	2.1	a	60.7	1.1	a	85.4	0.5	a
PBZ3W	32.1	1.7	a	61.6	1.7	a	84.2	0.3	a
PBZ4W	34.2	1.0	a	62.2	0.4	a	84.7	0.9	a

**Table 5:** Tuber weight and tuber number in seed-size categories of plants dehaulmed at 82 DAP. The results are the means of three replicates and normalized per mother tuber. Different letters indicate statistically significant differences (ANOVA, Tukey–Kramer’s HSD, *p* value ≤ 0.05). The means are the results of three replications. The standard error (SE) of the mean is indicated. The data are from Experiment 2. Weeks 1–4 are denoted as 1W to 4W.

**Tuber Weight at 82 DAP**												
**Tuber Width**	**35–45 mm**			**45–55 mm**			**55–60 mm**			**35–60 mm**		
**Treatment**	**Mean (kg)**	**SE** **(±)**	***p* Value ≤ 0.05**	**Mean (kg)**	**SE** **(±)**	***p* Value ≤ 0.05**	**Mean (kg)**	**SE** **(±)**	***p* Value ≤ 0.05**	**Mean (kg)**	**SE** **(±)**	***p* Value ≤ 0.05**
Control	0.10	0.018	d	0.30	0.020	d	0.31	0.017	a	0.72	0.021	bc
GA3	0.32	0.030	a	0.58	0.028	a	0.10	0.010	d	1.03	0.066	a
GA3–PBZ90 1W	0.24	0.037	abc	0.45	0.043	abc	0.07	0.010	d	0.79	0.092	abc
GA3–PBZ90 4W	0.29	0.036	a	0.50	0.019	abc	0.09	0.012	d	0.92	0.019	abc
GA3–PBZ270 1W	0.25	0.036	ab	0.44	0.060	abcd	0.10	0.004	d	0.84	0.092	abc
GA3–PBZ270 4W	0.27	0.033	a	0.54	0.037	ab	0.13	0.026	cd	0.97	0.066	ab
PBZ90 1W	0.11	0.019	d	0.35	0.005	cd	0.23	0.020	ab	0.71	0.016	bc
PBZ90 4W	0.11	0.011	cd	0.34	0.020	cd	0.26	0.032	ab	0.74	0.035	abc
PBZ270 1W	0.12	0.013	bcd	0.40	0.039	bcd	0.22	0.012	bc	0.76	0.062	abc
PBZ270 4W	0.09	0.001	d	0.30	0.018	d	0.25	0.006	ab	0.65	0.020	c
**Tuber number at 82 DAP**												
**Tuber width**	**35–45 mm**			**45–55 mm**			**55–60 mm**			**35–60 mm**		
**Treatment**	**Mean**	**SE** **(±)**	***p* value ≤ 0.05**	**Mean**	**SE** **(±)**	***p* value ≤ 0.05**	**Mean**	**SE** **(±)**	***p* value ≤ 0.05**	**Mean**	**SE** **(±)**	***p* value ≤ 0.05**
Control	2.38	0.440	cd	3.87	0.248	b	2.75	0.113	a	9.00	0.558	ab
GA3	5.99	0.625	a	6.04	0.247	a	0.69	0.063	c	12.71	0.860	a
GA3–PBZ90 1W	4.62	0.730	abc	4.57	0.446	ab	0.46	0.068	c	9.65	1.233	ab
GA3–PBZ90 4W	5.68	0.606	a	5.34	0.109	ab	0.65	0.081	c	11.67	0.479	ab
GA3–PBZ270 1W	4.75	0.634	abc	4.47	0.610	ab	0.66	0.041	c	9.89	1.132	ab
GA3–PBZ270 4W	5.27	0.541	ab	5.73	0.414	a	1.02	0.200		12.03	0.888	ab
PBZ90 1W	2.62	0.462	cd	4.39	0.052	ab	2.04	0.166	b	9.05	0.347	ab
PBZ90 4W	2.77	0.283	bcd	4.56	0.254	ab	2.34	0.283	ab	9.66	0.509	ab
PBZ270 1W	3.06	0.293	bcd	5.11	0.503	ab	1.89	0.103	b	10.07	0.849	ab
PBZ270 4W	2.06	0.020	d	3.84	0.219	b	2.27	0.068	ab	8.18	0.271	b

**Table 6:** Seed-size tuber lengths. Tubers of sizes 35–60 mm were measured for their length. Tubers from three dehaulming times were tested. Different letters indicate statistically significant differences (ANOVA, Tukey–Kramer’s HSD, *p* value ≤ 0.05). The means are the results of three replications. The standard error (SE) of the mean is indicated. The data are from Experiment 2. Weeks 1–4 are denoted as 1W to 4W.

	Tuber Length of 35–60 mm Seed-Size Tubers								
	82 DAP			95 DAP			103 DAP		
Treatments	Mean Length (mm)	SE (±)	*p* Value ≤ 0.05	Mean Length (mm)	SE (±)	*p* Value ≤ 0.05	Mean Length (mm)	SE (±)	*p* Value ≤ 0.05
Control	65.6	1.4	b	65.6	0.2	b	63.3	0.6	b
GA3	74.1	0.5	a	77.7	0.7	a	76.9	0.5	a
GA3–PBZ90–1W	74.2	0.5	a	77.4	1.0	a	76.8	0.4	a
GA3–PBZ90–4W	72.9	0.7	a	77.0	0.4	a	77.2	0.8	a
GA3–PBZ270–1W	74.6	0.6	a	78.4	1.0	a	77.0	0.5	a
GA3–PBZ270–4W	73.2	0.1	a	76.3	0.3	a	76.8	0.5	a
PBZ90–1W	64.1	0.8	b	64.9	0.5	b	62.7	0.7	b
PBZ90–4W	63.8	0.7	b	65.4	0.3	b	63.5	0.3	b
PBZ270–1W	62.8	0.1	b	63.2	0.3	b	62.5	0.2	b
PBZ270–4W	65.4	0.3	b	66.0	0.6	b	63.5	0.4	b

## Data Availability

The datasets generated during and/or analyzed during the current study are available from the corresponding author upon reasonable request.

## Supplementary Materials

The following supporting information can be downloaded at https://www.mdpi.com/article/10.3390/plants13162327/s1, Figure S1: Graphical representation of the treatments; Figure S2: Progressive growth and monitoring of potato plants; Figure S3: Dry canopy weight and number of stems; Table S1: Tuber weight and number at 95 DAP; Table S2: Tuber weight and number at 103 DAP.

## Abbreviations

DAP	Days after planting
GA	Gibberellic acid
PBZ	Paclobutrazol
