# Supplementary material for: The Application of Paclobutrazol to GA3-Treated Seed Tuber Potato Fields Does Not Shorten the Growth Cycle or Mitigate Tuber Elongation
Source: Plants (Basel). 2024 Aug 21;13(16):2327. doi: 10.3390/plants13162327 (PMC11359537; doi:10.3390/plants13162327)
Supplement: Supplementary file 1 [file plants-13-02327-s001.zip › plants-3052357-supplementary.pdf]

Supplementary Materials

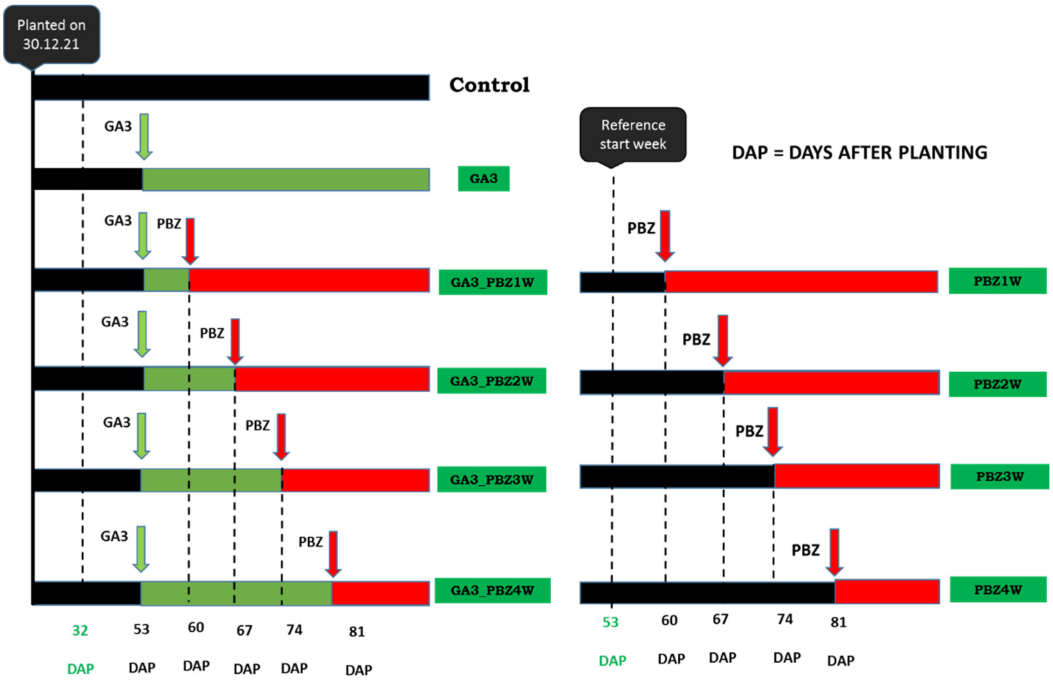

Figure S1. Graphical representation of the treatments applied in experiment 1. Black bars indicate continuous uninterrupted growth of control plants. Green arrows indicate GA_3 application and green bars are growth after GA_3 application. Red arrows indicate PBZ application and red bars growth after PBZ application. GA_3 was applied at 53 days after planting (DAP). PBZ was applied in one week intervals from the time of GA_3 application for four weeks.

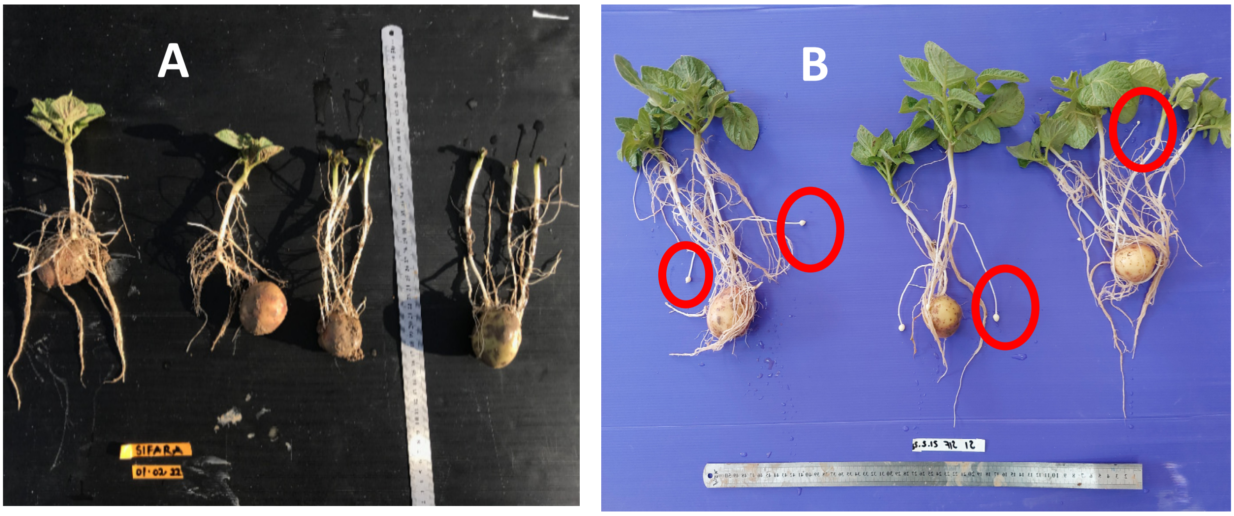

Figure S2. Progressive growth and monitoring of potato plants before and at treatment application. The image to the left is the tracking of uniform plant emergence in the field (A). The image to the right is the growth stage, with initial tuberization of tubers less than 0.5 cm in diameter (red circled) at the time at which the application treatments started (B).

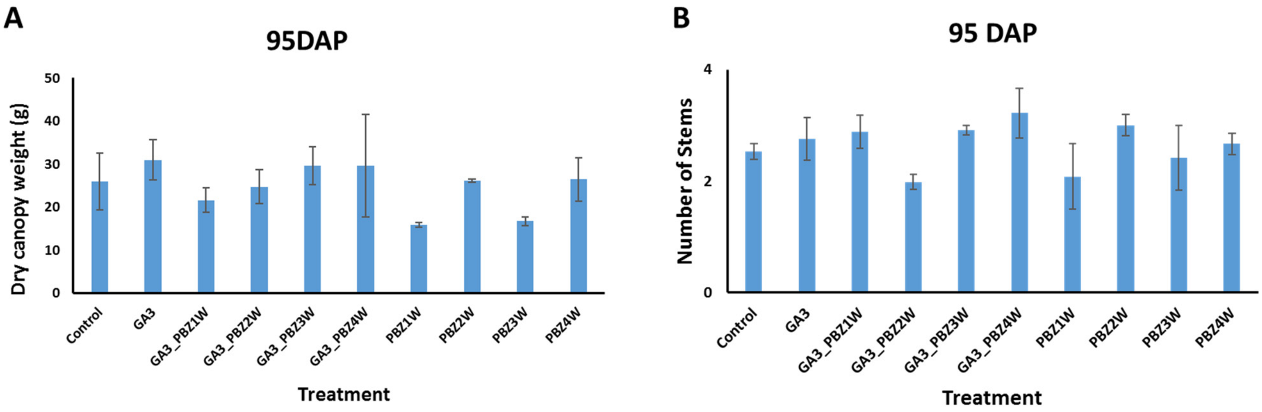

Figure S3. Dry canopy weight and number of stems at 95 days after planting: (**A**). Values for dry canopy weight, given as means normalized by the number of stems measured per replicate and standard error bars of three replicates per treatment; and (**B**). Number of stems normalized per mother tuber. Columns with error bars not marked with letters show no significant difference (ANOVA Tukey–Kramer HSD, Pval ≤0.05).

Table S1. Tuber weight and number at 95 DAP. Tuber weight and tuber number in individual seed size categories or all seed size categories (35–60 mm) of dehaulmed plants at 95 DAP. Results are means of three replicates and normalized per mother tuber. Different letters indicate statistically significant differences (ANOVA Tukey–Kramer HSD, Pval ≤0.05). Standard error (SE) of mean is indicated.

Tuber weight at 95 DAP

**Tuber width**	**35–45 mm**			**45–55 mm**			**55–60 mm**			**35–60 mm**		
**Treatment**	Mean (kg)	SE (±)	P value <0.05	Mean (kg)	SE (±)	P value <0.05	Mean (kg)	SE (±)	P value <0.05	Mean (kg)	SE (±)	P value <0.05
**Control**	0.06	0.008	b	0.24	0.052	b	0.21	0.014	a	0.51	0.074	b
**GA3**	0.25	0.018	a	0.68	0.042	a	0.29	0.046	a	1.22	0.022	a
**GA3_PBZ90 1W**	0.20	0.012	a	0.54	0.030	a	0.29	0.013	a	1.03	0.034	a
**GA3_PBZ90 4W**	0.20	0.026	a	0.60	0.033	a	0.28	0.053	a	1.08	0.106	a
**GA3_PBZ270 1W**	0.19	0.029	a	0.58	0.046	a	0.33	0.022	a	1.10	0.061	a
**GA3_PBZ270 4W**	0.24	0.019	a	0.64	0.008	a	0.29	0.010	a	1.17	0.024	a
**PBZ90 1W**	0.08	0.006	b	0.28	0.037	b	0.26	0.009	a	0.62	0.027	b
**PBZ90 4W**	0.07	0.007	b	0.24	0.019	b	0.28	0.004	a	0.58	0.021	b
**PBZ270 1W**	0.08	0.006	b	0.28	0.029	b	0.23	0.020	a	0.59	0.053	b
**PBZ270 4W**	0.07	0.007	b	0.28	0.018	b	0.29	0.031	a	0.65	0.051	b

Tuber number at 95 DAP

**Tuber width**	**35–45 mm**			**45–55 mm**			**55–60 mm**			**35–60 mm**		
**Treatment**	Number	SE (±)	P value <0.05	Number	SE (±)	P value <0.05	Number	SE (±)	P value <0.05	Number	SE (±)	P value <0.05
**Control**	1.45	0.188	b	2.97	0.656	c	1.87	0.144	a	4.77	0.520	b
**GA3**	4.74	0.336	a	6.86	0.429	a	1.99	0.343	a	11.48	0.412	a
**GA3_PBZ90 1W**	3.93	0.277	a	5.33	0.236	ab	1.98	0.107	a	9.84	0.595	a
**GA3_PBZ90 4W**	3.96	0.462	a	6.06	0.362	a	1.94	0.347	a	9.86	1.252	a
**GA3_PBZ270 1W**	3.67	0.468	a	5.73	0.490	a	2.17	0.098	a	9.52	0.857	a
**GA3_PBZ270 4W**	4.81	0.355	a	6.52	0.141	a	1.98	0.096	a	11.60	0.680	a
**PBZ90 1W**	1.86	0.170	b	3.60	0.444	bc	2.23	0.078	a	5.95	0.413	b
**PBZ90 4W**	1.64	0.156	b	3.03	0.251	c	2.51	0.011	a	5.80	0.304	b
**PBZ270 1W**	2.07	0.137	b	3.58	0.365	bc	2.05	0.177	a	6.19	0.441	b
**PBZ270 4W**	1.63	0.175	b	3.67	0.187	bc	2.58	0.248	a	5.84	0.512	b

Table S2. Tuber weight and number at 103 DAP. Tuber weight and tuber number in individual seed size categories or all seed size categories (35–60 mm) of dehaulmed plants at 103 DAP. Results are means of three replicates and normalized per mother tuber. Different letters indicate statistically significant differences (ANOVA Tukey–Kramer HSD, Pval ≤0.05). Standard error (SE) of mean is indicated.

Tuber weight at 103 DAP

Tuber width	35–45 mm			45–55 mm			55–60 mm			35–60 mm		
**Treatment**	Mean (kg)	SE (±)	P value <0.05	Mean (kg)	SE (±)	P value <0.05	Mean (kg)	SE (±)	P value <0.05	Mean (kg)	SE (±)	P value <0.05
**Control**	0.06	0.010	c	0.25	0.038	b	0.24	0.029	b	0.55	0.071	b
**GA3**	0.14	0.025	ab	0.59	0.060	a	0.57	0.054	a	1.30	0.131	a
**GA3_PBZ90 1W**	0.16	0.007	a	0.53	0.046	a	0.37	0.015	ab	1.05	0.051	a
**GA3_PBZ90 4W**	0.16	0.016	a	0.58	0.043	a	0.49	0.092	a	1.24	0.084	a
**GA3_PBZ270 1W**	0.13	0.005	ab	0.51	0.032	a	0.41	0.017	ab	1.04	0.035	a
**GA3_PBZ270 4W**	0.18	0.018	a	0.64	0.050		0.51	0.025	a	1.32	0.056	a
**PBZ90 1W**	0.09	0.002	bc	0.27	0.013	b	0.25	0.052	b	0.61	0.065	b
**PBZ90 4W**	0.08	0.012	bc	0.24	0.028	b	0.25	0.025	b	0.57	0.065	b
**PBZ270 1W**	0.09	0.005	bc	0.29	0.021	b	0.28	0.027	b	0.65	0.021	b
**PBZ270 4W**	0.08	0.004	bc	0.26	0.034	b	0.27	0.023	b	0.60	0.060	b

Tuber number at 103 DAP

Tuber width	35–45 mm			45–55 mm			55–60 mm			35–60 mm		
**Treatment**	Number	SE (±)	P value <0.05	Number	SE (±)	P value <0.05	Number	SE (±)	P value <0.05	Number	SE (±)	P value <0.05
**Control**	1.52	0.215	d	3.22	0.449	c	2.20	0.254	b	6.94	0.839	c
**GA$_3$**	2.79	0.477	abc	6.33	0.655	a	4.18	0.414	a	13.30	1.498	a
**GAPBZ90 1W**	3.07	0.147	abc	5.44	0.454	ab	2.52	0.098	b	11.04	0.527	ab
**GAPBZ90 4W**	3.37	0.282	ab	6.06	0.535	a	3.47	0.677	ab	12.89	0.848	a
**GAPBZ270 1W**	2.57	0.137	abcd	5.16	0.355	abc	2.89	0.118	ab	10.62	0.424	abc
**GAPBZ270 4W**	3.57	0.322	a	6.64	0.521	a	3.60	0.181	ab	13.82	0.736	a
**PBZ90 1W**	2.12	0.049	cd	3.52	0.197	bc	2.24	0.440	b	7.88	0.644	bc
**PBZ90 4W**	1.93	0.314	cd	3.14	0.377	c	2.26	0.234	b	7.33	0.917	bc
**PBZ270 1W**	2.14	0.118	bcd	3.77	0.214	bc	2.41	0.229	b	8.32	0.245	bc
**PBZ270 4W**	2.02	0.088	cd	3.33	0.426	bc	2.38	0.191	b	7.73	0.695	bc
